# Factors Favoring and Hindering Volunteering by Older Adults and Their Relationship with Subjective Well-Being: A Mixed-Method Approach

**DOI:** 10.3390/ijerph18136704

**Published:** 2021-06-22

**Authors:** Eva Serrat-Graboleda, Mònica González-Carrasco, Ferran Casas Aznar, Sara Malo Cerrato, David Cámara Liebana, Marta Roqueta-Vall-Llosera

**Affiliations:** 1 Faculty of Nursing, University of Girona, 17004 Girona, Spain; david.camara@udg.edu (D.C.L.); marta.roqueta@udg.edu (M.R.-V.-L.); 2 Nurse in Parc Hospitalari Martí i Julià, 17190 Girona, Spain; 3 Faculty of Education and Psychology, University of Girona, 17004 Girona, Spain; monica.gonzalez@udg.edu (M.G.-C.); ferran.casas@udg.edu (F.C.A.); sara.malo@udg.edu (S.M.C.); 4 Midwife in Hospital Universitari Josep Trueta, 17007 Girona, Spain

**Keywords:** volunteering, subjective well-being, older adults, predictive factors, mixed methodology

## Abstract

A mixed methodology was used through the triangulation of quantitative and qualitative data to determine older adults’ perspectives regarding volunteering and identify what factors can contribute to promoting it, with special emphasis on the role that their own well-being plays in this behavior. The results reveal that satisfaction with life as a whole contributes positively to volunteer behavior and satisfaction with the groups one belongs to contributes negatively. The volunteers were less satisfied than non-volunteers with interpersonal relationships and with the groups they belong to. Knowing the opinion of the older adults with regard to volunteering and understanding how this prosocial behavior relates to their own well-being is very useful for developing strategic plans that allow future volunteers to be captured.

## 1. Introduction

Often, old age is still related to unproductivity, dependency, or loss in different areas of life [1,2,3] aspects that project an image of a vulnerable group or social difficulties in older people despite the resources they can contribute to society. By contrast, Butler (2000, cited in [2]) defined productive aging as “the ability of an individual or population to serve by doing paid work, volunteer activities, helping the family and remaining active in any way” [2] (p. 140). This concept of aging is framed within activity theory, which argues in favor of the importance of continuing activities done prior to retirement [4,5], as well as initiating new activities, including volunteer tasks.

In this study, the concept of productive aging is taken as a basis for understanding the role volunteering can play in older people’s well-being, understanding volunteering as referring “to a wide range of activities including mainstream forms of mutual support and self-help, formal service provision and other forms of civic engagement, done voluntarily, for the benefit of society as a whole and without economic retribution being the main motivating factor” [6] (p. 3).

Of the different types of volunteering that are contemplated in the Spanish legislation, we have focused on the socio-health sector which includes the following: “The promotion of health, the prevention of illness, health care, rehabilitation and social care aimed at society as a whole or vulnerable groups, and which, through comprehensive and specialized intervention in relation to physical, psychological and social aspects, offers support and guidance to families and those closest to them, improving living conditions” [7] (p. 7).

With regard to personality factors linked to volunteering, Binder and Freytag [8] found a significant relationship between being open and extroverted and a greater predisposition towards doing volunteer work. In this case, 5000 British households were studied through 15,000 individual interviews, the sample consisting of people aged over 16. In another study, Okun, Pugliese, and Rook [9] surveyed 888 adults aged from 65 to 90 and observed that being in contact with friends or going to centers (clubs) or organizations favored the fact of being a volunteer, and that extroverts tended to go to these centers more and have relationships with a larger number of people. Based on the Big Five model, a Spanish language study conducted by Ledesma, Sánchez, and Díaz-Lázaro [10] proposed a list of 67 Adjectives to Evaluate Personality (AEP) to the Argentine population, with a sample of ages between 18 and 80 years old. These authors created the list to compensate for the difficulties involved in translating or contextualizing the subcategories proposed by the Big Five model in other languages. In a later study, Sánchez and Ledesma [11] retested the AEP on an Argentine population aged between 18 and 89, also obtaining adequate psychometric properties.

Although some studies have linked certain personality traits to the act of volunteering, we do not know of any research that explores perceived characteristics or adjectives like those proposed in the AEP (such as being polite, generous, etc.), that is, beyond personality characteristics, and related them to the volunteer’s well-being, which we believe to be a novel contribution of this study.

Factors that have been identified as hindering volunteering in formal organizations include a lack of time or interest and health problems [12]. Psychological distance, understood as the perception that formal organizations are overly bureaucratic and complicated, has also been found to be a hindering factor regardless of age [13].

A bibliographical review carried out by Dávila de León and Díaz–Morales [14] highlighted the following as factors that favor volunteering work among the older adults: a good economic level, a high level of education, good health, the belief that they will improve and expand their social relations, and the perception of receiving social support when performing this type of work.

There are also a number of obstacles to older adults doing volunteer work. In the study conducted on a sample of 735 people aged over 65 by Medina–Tornero and Carbonell–Cutillas [15], 71.5% of the volunteers surveyed and 73.8% of the non-volunteers expressed agreement with the statement “Older adults have time to volunteer, but there are almost no programmes or actions for them to participate in” [15] (p. 34). The study presented here explored the factors that facilitate and hinder social-health volunteering from the perspective of the older adults themselves. A mixed methodology has been used, which to the best of our knowledge has not been done in other investigations.

Several studies highlight the physiological and mental health benefits of volunteering [14,15,16]. In addition, for the older adults, volunteering also means an improvement in their role and social image [16]. A positive correlation has been observed between a higher quality of life and volunteering in the older adults, although a cause–effect relationship has not been established. De Souza, Lautert, and Hilleshein [17] found that there was a statistically significant and positive correlation between volunteering and the psychological and social domains in the reduced version of the World Health Organization Quality of Life Scale (Whoqol-brief).

According to the study carried out by Gil–Lacruz et al. [18], countries with a high rate of older people involved in volunteering activities also had higher health rates (Sweden) compared to countries with lower rates of volunteering, which had the lowest health indices (Eastern European countries). In terms of physiological health, recent studies have shown that helping others can have the benefits of improving chronic inflammation and quality of sleep [19]. Volunteering among older people is also positively related to greater well-being and lower prevalence of depression [20]. What is more, volunteering also has positive repercussions on loneliness [21] and cognitive functioning [22,23,24,25] among older people, and lowers the risk of dementia [26]. Specifically, both informal caregiving tasks such as babysitting and volunteering were found to be associated with lower cognitive decline among older adults over a two-year period [27].

In our study, the focus was on the subjective side of quality of life, which is generally defined as subjective well-being, since our objective is to determine the perceptions, evaluations, and opinions of the older adults themselves regarding volunteering given the scarcity of studies on this subject.

The correlation between volunteering and well-being has been explored in previous studies. For example, the more time spent volunteering and the greater the frequency of it, the higher an individual’s subjective well-being [13]. Binder and Freig [8] and Meier and Stutzer [28] also argued that continuing to volunteer on a regular basis increases subjective well-being. In addition, Mellor et al. [29] observed a positive correlation between volunteering and the Neighbourhood Well-Being Index (NWI) and Personal Well-Being Index (PWI), regardless of age group (the sample included people of Australian nationality aged 18 to 88) or geographical location (rural or urban).

No studies have been found in the literature that explore the relationship between the different satisfaction domains comprising the Personal Well-Being Index (PWI) and doing volunteer work. Although the study by Mellor et al. [29] did consider the Personal Well-Being Index, it did not distinguish between satisfaction domains. The present study intended to explore this issue in order to carry out an in-depth analysis of the productive aging process and its relationship with volunteering in the Spanish context.

The aim of this study was, therefore, to carry out the aforementioned analysis by determining the perceptions, evaluations, and opinions of the older adults themselves regarding volunteering, as well as identifying predictive, hindering. and facilitating variables in this respect, with special emphasis on the role satisfaction with specific life domains plays with regard to this behavior, an aspect yet to be explored in the scientific literature, as far as we are aware.

This article took as a starting point the consideration that volunteering is positively related to higher well-being among older volunteers. Specifically, it is expected that being satisfied with health, with interpersonal relationships, and having people who have volunteered in one’s social network would be the most important variables when predicting a willingness to volunteer. We also expected that the AEP adjectives considered in this study would favor the involvement of older adults in volunteering tasks, and finally, that poor perception of health, lack of time, and difficulties in accessing information about volunteering would be factors that make it difficult for older adults to get involved in this type of activity. The use of a mixed methodology through the triangulation of quantitative and qualitative data allowed us to obtain complementary data on the research problem, comparing and contrasting the data obtained using each of the two methodologies provides the research with greater validity and a better understanding of the phenomenon [30]. Specifically, the questionnaire allows for the systematic and structured collection of data provided by a substantial number of informants, while incorporating an introspective component, as the informant must reflect in order to respond to the questions posed [31]. On the other hand, the focus group allowed us to gather information on a previously defined topic based on personal experiences; although the group was relatively small, the information gathered was highly in-depth [32].

## 2. Method

### 2.1. Participants

The population under study were older adults who attended two senior citizens’ activity clubs (*casals*) located in the different territorial contexts of Barcelona city and Besalú (a province of Girona, Northeast Spain), the aim being to determine their different perspectives and views in relation to carrying out volunteering activities. The sample was selected using the non-probability method by convenience, i.e., users of the two selected centers were asked if they wished to participate voluntarily in the study in person.

These two senior citizens’ activity clubs were selected because of the support they offered to carry out the study. It was considered appropriate to use senior citizens’ activity clubs as an access route because it is a less invasive way of reaching the over-65-year-olds than conducting a home survey. 

A senior citizens’ activity club is a civic facility for this age group aimed at promoting their well-being and participation as active members of society [33]. Participants must be 60 or over or be a pensioner or unemployed and aged over 52. Attendees’ spouses or partners can also access the senior citizens’ activity club, regardless of age [33]. It is for this reason that although the initial proposal was to study people aged over 65, people who did not fit in this age group were ultimately accepted because they went to the selected senior citizens’ activity clubs. The questionnaire was answered by a total of 85 people at the two senior citizens’ activity club M = 71.18 years; *dt* = 7.538; age range = 59–86) and a total of 21 people from the two senior citizens’ activity club participated in discussion groups.

### 2.2. Instruments

Two data collection techniques were used: a questionnaire and a semi-structured script for the discussion groups administered in that order. The questionnaire had a self-administration format and was used to explore variables related to volunteering.

In addition, the discussion group was chosen as a data collection method because it is especially useful for “discovering people’s perception of what generates or prevents a behaviour, as well as their reaction to different ideas, behaviours, products or services” to “identify personal and community needs” and because it can be used as part of a mixed research methodology [34] (p. 53). In our study, we were able to collect data on the perceptions of older adults regarding the motivating and hindering factors related to volunteering and identify what actions would be necessary to promote it.

Both the questions from the questionnaire and the focus group are displayed in Table 1.

### 2.3. Procedure

The study was correlational and cross-sectional in design, with an exploratory nature given the scarcity of studies of this type. It consisted of several sessions in which a questionnaire was administered and a discussion group organized in each participating senior citizens’ activity club. The procedure for administering the questionnaire was different in each senior citizens’ activity club, in order to respect their respective dynamics. This also allowed us to determine the advantages and disadvantages of each procedure.

In the Horta senior citizens’ activity club (H) (The quotes pertaining to the discussion group held at the Horta senior citizens’ activity club (Barcelona) will henceforth be identified with an H, and those from the discussion group held at the Besalú senior citizens’ activity club (Girona) with a B.), the questionnaire was administered to those who were present on a single day, previously specified, and the facilitator was responsible for resolving any doubts that arose; in the Besalú senior citizens’ activity club (B), a person from the senior citizens’ activity club, after training by the researchers, took responsibility for the questionnaires and sent them to those attending the senior citizens’ activity club over several days, and it was this same person who was responsible for resolving the doubts of the older adults.

A discussion group was held in each of the two participating senior citizens’ activity clubs, lasting approximately one hour, and was conducted by the researchers. In the Horta senior citizens’ activity club, 8 people initially participated (5 volunteers and 3 non-volunteers), but during the activity more people were added until there were finally 13). In the Besalú senior citizens’ activity club, 8 people took part (6 volunteers and 2 non-volunteers).

## 3. Data Analysis

With the quantitative data obtained through the questionnaire, bivariate statistical analyses were performed between the variables described in the instruments section and the fact of having done volunteer work, calculating the chi-square (χ^2^) or comparing means with the Student’s *t*-statistic (t) depending on the type of variable studied, together with the size of the effect when the result was statistically significant. In addition, a nonparametric test (the Mann–Whitney U test) was applied when the subgroup under consideration had fewer than 30 participants.

In the data analysis, no distinction was made between those participants who only volunteered in the past and those who continued to volunteer at the time of administering the questionnaire after verifying that they did not present statistically significant differences in any of the variables studied.

A binary logistic regression was also calculated, having done tasks as a volunteer being considered as a dependent variable and the satisfaction domains included in the PWI and the item of satisfaction with life as a whole being considered as independent variables. The rationale for choosing this technique was that logistic regression is a powerful tool that allows multiple explanatory variables to be analyzed simultaneously, while at the same time reducing the effect of confounding factors [45].

Statistical package SPSS v.23 was used for all of the above.

The analysis of the data obtained through the discussion groups was performed using the NVivo8 program. After obtaining a literal transcription, the content of each of the questions was carefully analyzed for coincidences and discrepancies in order to identify a category of analysis that included the different points of view and was relevant in terms of the pre-established aims. The categories of analysis were discussed and agreed upon within the research team, and those that did not reach a consensus of at least 80% were discarded.

Finally, the data were integrated through multi-method triangulation, which consisted of at least two types of triangulation (combining methods, data, researchers and/or theories) [46]. In this study, we employed data triangulation and triangulation between methods, using the data from the two discussion groups and responses to 85 questionnaires, and a questionnaire and a semi-structured script for the discussion groups, respectively. While all of these methods have weaknesses, this type of triangulation allowed them to be compensated for, thus contributing greater validity to the research [46].

## 4. Ethical Considerations

Prior authorization was obtained from the Catalan Government’s Department of Health, given that this was the department that requested and funded the research. Since the participants were adults, they themselves gave their verbal consent to participate in the study before answering the questionnaire and/or participating in the focus group.

## 5. Results

Due to the low percentage of volunteers identified, it was not possible to carry out a separate analysis for the two senior citizens’ activity clubs, so the results below refer to the total number of questionnaires answered. However, no statistically significant differences were observed between the two senior citizens’ activity club regarding the fact of having carried out volunteering activities or not. With regard to the information provided by the discussion groups, this is detailed in the text, specifying the senior citizens’ activity club at which it was discussed.

### 5.1. Volunteering among the Older Adults

Of the 85 people who responded to the questionnaire, more than half (53.7%) said they did not know of any organization associated with volunteering, something the participants themselves considered a factor that makes it difficult to recruit volunteers: “There is a lack of information regarding volunteering and the work that can be done, and a lack of rewards” (B). Of those they were aware of, the best known were FATEC (the largest older adults association in Catalonia), Caritas (an NGO that fights poverty and social exclusion), and the senior citizens’ activity club closest to the area where they lived. However, the following opinion was also identified: “There are many associations to volunteer with, and anyone who wants to do this type of work knows this” (H).

Some 85% said they knew someone who volunteered (mainly friends, family, or acquaintances from the senior citizens’ activity club), this being a statistically significant factor with the person also being a volunteer ($\chi_{1}^{2}$ = 9315; *p* = 0.002; V = 0.355). Of the total sample, 37.6% (32 people) said they had done volunteer work, but only 20 of these responded to the specific section of the questionnaire addressed at people who had volunteered at some time. Of these, 16 answered affirmatively to the question: Are you currently doing volunteer work? A significant percentage (75.9%) of the total sample expressed knowledge of the type of work that older adults do in socio-health contexts. Discussion group participants emphasized that the volunteer work done by the older adults is mainly socio-cultural in nature and that little work is done in the health field (leading memory groups, etc.). Of the people who currently volunteered, only 31.3% did so in the social field (helping other people), the rest in the cultural sphere.

Some 80.6% agreed that older adults could help other people with their health and public health issues and 44% thought that the biggest contribution they can make is to spend time with others and keep them company. Other aspects that 16% mentioned as work that could be done were doing paperwork and helping the sick and family members. According to the opinions gathered in the discussion groups, other work that is often carried out by volunteers is administrative in nature and related to managing volunteer activities and overseeing the dynamics of the senior citizens’ activity club where volunteering is carried out.

### 5.2. Perceived Characteristics of Volunteers

The characteristics the participants valued as most important in volunteers were as follows: being polite, being able to listen, being optimistic, and being supportive (Table 2). When analyzing how these characteristics related to the person having been a volunteer or not, the only ones to emerge as significant were being happy (t_(61)_ = −2598; *p* = 0.012; η_p_^2^ = 0.1), the higher mean being among the group of people who had been volunteers (M = 4.42, SD = 0.848) compared to those who had not (M = 3.81, SD = 0.988); and being a good person (t_(59_._688)_ = −2103; *p* = 0.040; η_p_^2^ = 0.065), the mean also being higher among the group of people who had volunteered compared to those who had not (M = 4.55, SD = 0.783 versus M = 4.06, SD = 1.071).

The participants in the discussion groups stated that it is necessary for the volunteer to have an “open mind” (H), be “tolerant” (H) and “altruistic and patient” (H), “have patience and take things slowly” (B), “have the capacity to adapt to the situation you are in” (B), “understand others and be polite” (B), and also feel useful and rewarded for what they do. They highlighted as important factors the “life optimism” (B) and “internal motivations” (B) of the individual and believed that due to this last factor it is often difficult to attract volunteers because many older people are exclusively looking for fun. In addition to all the qualities mentioned above, they believed that “good specific training” is required in certain types of help (H).

### 5.3. Factors That Facilitate and Hinder Volunteering

Table 3 shows the order of importance of factors that facilitate volunteering among those proposed to the over-65-year-olds, according to the questionnaire responses provided by the older adults participants in our study. Among these factors, they especially emphasized being in good health and having time; the latter was also mentioned in the discussion groups as “having time available” (B). None of the studied factors was statistically significant in relation to the fact of being or having been a volunteer or not.

They also clearly differentiated between volunteering in the field of health outside the home and taking care of a family member. In this sense, they stated that “taking care of a person at home is more sacrifice and an obligation” (B), which requires a lot of patience and willpower, while “volunteering is a vocation” (B). The fact of having grandchildren or not (they considered babysitting grandchildren to be another type of volunteer work that is often abused) and having other family obligations emerged as hindering factors.

Respondents who did or had ever done volunteer work rated the local government’s promotion of volunteering quite low (2.32 out of 5), “the authorities do not sufficiently promote volunteering” (H). They considered the main source of support or assistance they receive or have received to be from other volunteers, family or friends, or the volunteering organization, rating the support they receive to do volunteer work 7.26 out of 10. Some 55.6% stressed that in order to promote volunteer work among the older adults the most important thing is to provide more information and disseminate it more: “to give informative talks on volunteering” (H) that motivate people to join and “orientation and/or training talks” (H) on how volunteer work can be done appropriately; and to be able to “obtain some type of benefit even if it is not economic” (H). However, some participants were opposed to this and considered that volunteering should not be rewarded in any way. Participants also stressed that it would be good to “have spaces for exchange” (H) between volunteers and the different volunteer associations.

### 5.4. The Relationship between Volunteering, Well-Being, and Health

Those people who reported being or having been a volunteer displayed a high level of satisfaction with doing or having done this type of work (8.37 out of 10). Volunteers stated that such work had the following main consequences for them: personal satisfaction (31.3%), which allowed them to help others (25%): “anyone who helps others is helping themselves” (B), increased self-esteem (12.5%), and “feeling useful” (B). When asked for further positive consequences, they mentioned meeting other people (57.1%). As for negative consequences, more than half (66.7%) expressed sadness over having to see the needs of others. Some participants in the discussion groups reported that seeing ill people can be depressing for the volunteer; and 33.3% of those who had volunteered highlighted the little recognition they had received.

In the discussion groups, the participants considered that volunteering is closely linked to well-being and health, “volunteering brings well-being, is rewarding and extends the life of those who do it” (B), to the extent that it is an important source of personal satisfaction that allows them to experience productive aging.

If we analyze well-being more closely, the areas in which they expressed the greatest satisfaction were interpersonal relationships, their life achievements, and the groups of people they belonged to; in last place came satisfaction with spirituality and/or religious beliefs, future security, and satisfaction with their own health (Table 4). Their overall life satisfaction had a mean score of 7.39 out of 10 for the whole sample: 8.12 among those who had volunteered and 6.76 among those who had not. It should be noted that although higher means were observed in almost all areas of satisfaction among those who had volunteered (except satisfaction with the groups they belonged to and satisfaction with interpersonal relationships), only with the single item on overall life satisfaction was the difference statistically significant.

Those respondents who reported being or having been a volunteer had a mean satisfaction of 8.37 out of 10 regarding this behavior; this result is consistent with the high satisfaction displayed by the participants in the discussion groups in relation to doing volunteer work. Among the advantages of doing this type of work, they highlighted being able to forget everything and get away from their own problems: “being useful and helping others is very rewarding” (H), and “discovering that there are many things to do for older people and that this is very satisfying” (H).

### 5.5. Explanatory Variables for Volunteering

Binary logistic regression analysis was used to identify explanatory variables for doing voluntary work among the older adults in the sample. A model was calculated based on the degree of satisfaction with the different well-being domains included in the PWI together with the item on overall life satisfaction.

The analyzed model had a good fit, as Table 5 shows, and a good classifying capacity (Table 6) according to the results of the Hosmer and Lemeshow test ($\chi_{8}^{2}\;$= 8087, *p* = 0.425).

Of all the variables included in the model, only 2 were significant (Table 7): one with a positive contribution (overall life satisfaction) and one with a negative contribution (satisfaction with the groups you belong to).

Table 8 summarizes the coincidences and discrepancies found when analyzing the responses separately between the two senior citizens’ activity clubs.

## 6. Discussion

This article describes the first study to have analyzed the perspectives of the older adults regarding volunteering and its connection with subjective well-being as a contribution to scientific knowledge on the subject, the aim being that it can be replicated with broader samples in the future. The use of a mixed methodology and data triangulation has compensated for the gaps in information that are sometimes produced when only analyzing quantitative data. Specifically, it has allowed a more in-depth interpretation and understanding of the issue under investigation.

### 6.1. Volunteering among the Older Adults

Of the total sample studied, 37.6% said they had done voluntary work, though only 16 people would like to continue with this type of work today. This participation rate in voluntary work is in line with the figure of 18% of older adults volunteers found in the study by Medina and Carbonell [15]. This important difference in results may be due to differences in the sampling and/or data collection instruments.

Some 85% of our sample reported knowing someone who volunteers (mainly friends, family, or acquaintances at the senior citizens’ activity club), this factor being related in a statistically significant way to the fact of also being a volunteer. This result confirms the important role played by social networks in favoring the older adults involvement in prosocial and voluntary activities, as Binder and Freytag [8] highlighted in their study. Our hypothesis that knowing people who volunteer among our sample would be a very important factor in becoming a volunteer was therefore fully confirmed.

### 6.2. Characteristics Perceived by Volunteers

Being a happy and good person are adjectives that the participants identified as most important when describing a volunteer, which brought our results close to those of Binder and Freytag [8]. Thus, in this regard, only our hypothesis that two of the adjectives considered in the AEP list in this study would favor the involvement of older people in volunteering was confirmed, as the other analyzed adjectives were not statistically significant.

### 6.3. Facilitating and Hindering Factors in Volunteering

As hindering factors, we found a lack of good health and family obligations, as well as a lack of training and institutional support. Participants in our study believed that the older adults involvement in voluntary work could be improved through interventions in these latter two factors. These data are congruent with those found in the studies by Agulló et al. [16], and Medina and Carbonell [15]. Taking into account that volunteering has a positive impact on health and well-being among the older adults [14,15,16,17], plus a positive impact at a social level [16], it should be promoted as an activity by public bodies. However, those respondents who volunteer or have been a volunteer are critical regarding the volunteering promoted by public administrations and believe there is a need for more information and increased publicity to improve older adults’ involvement in social work. Family obligations have emerged as a factor that hinders volunteering by older adults, as these deprive them of time. Therefore, our hypothesis confirms and supports the results found in other studies. It also confirms that poor health perception and poor information about volunteering is negatively related to older people’s involvement in volunteering.

In the study by Medina and Carbonell [15], 71.5% of the volunteers surveyed and 73.8% of the non-volunteers emphasized the perception of there being few options to participate in voluntary work; similar results were reflected in our study through the low score (2.32 out of 5) that respondents who were or had been volunteers gave for the promotion of voluntary work by public administrations in Spain and the perception of discussion group participants that it is not promoted sufficiently. These data suggest that if actions were carried out to promote and disseminate voluntary work, the older adults’ involvement may well increase.

### 6.4. The Relationship between Volunteering, Well-Being, and Health

Both in the discussion groups and the responses to the questionnaires, a higher feeling of satisfaction was observed among the people who do or have done volunteer work in the different well-being domains studied, although this difference is only significant in the case of overall life satisfaction. These results are in line with others in the scientific literature, and many studies have found a significant relationship between physical and mental health and doing volunteer work [14,15,16], while others have observed one between life satisfaction and well-being [8,17,29].

This article makes an important contribution to knowledge on the subject because it analyzes the relationship between volunteering and different domains of subjective well-being. The domains in which those who currently do volunteer work or have done it in the past show the greatest satisfaction are: (1) interpersonal relationships, (2) things they have achieved in life, and (3) perceived present safety. However, it should be noted that the volunteers in our sample (in the past and present) are less satisfied than the non-volunteers in two of these domains, specifically interpersonal relationships and perceived present safety. Despite these differences not being statistically significant in any of the cases, these data contradict what one would intuitively expect and suggest that many people may become volunteers seeking more satisfaction in their interpersonal relationships. Perhaps volunteers do not depend on or only seek more interpersonal relationships at a quantitative level as shown in the study by Prouteau and Wolff [47], but rather their real motivation in volunteering is to improve their relationships on a qualitative level because they are not satisfied with the ones they have in their environment. Thus, our initial hypothesis that volunteers would be more satisfied with their personal relationships is partially rejected. On the one hand, having an extensive social network favors volunteering as discussed above; however, the older volunteer would not be satisfied with his or her relationships and would seek to enhance and improve them qualitatively by carrying out volunteering tasks.

### 6.5. Explanatory Variables of Volunteering

As explanatory factors for volunteering behavior, after analyzing the variables included in the logistic regression model we observed that satisfaction with life as a whole contributes positively to volunteer behavior and satisfaction with the groups you belong to contributes negatively. The fact that people who are satisfied with their life overall participate more in volunteer activities could be interpreted as their feeling good driving them to want to help others. The fact that people who are already satisfied with the groups they belong to become less involved in volunteer activities might be because they do not think these activities could increase the satisfaction they already enjoy. Our results make an important and novel contribution to knowledge in this area because although facilitating and motivational factors are identified in the current literature, in the bibliographic review by Dávila de León and Díaz–Morales [14], for example, satisfaction with different life domains, and consequently their relationship with well-being, are not taken into account.

## 7. Conclusions

By way of conclusion, doing supportive activities with others as a volunteer affects the well-being of the volunteer, and it is therefore important to do everything possible to promote it. Having a greater knowledge of the older adults’ perspective on these matters, as well as knowing which factors are relevant to them, would allow actions to be developed that promote volunteering among this group.

## 8. Practical Implications

From the results obtained, we can see how important it is for health and social intervention professionals to inform and try to involve older people in volunteer work or prosocial behavior, as, according to our results, these behaviors have benefits for the well-being of older volunteers.

## 9. Limitations

An important limitation of this study was the impossibility of establishing cause–effect relationships between the variables studied, which would require randomly assigning older people to experience volunteering and comparing the outcomes of this experience to the feelings of those who do not volunteer.

Another limitation of the study was the reduced sample size. The fact of having chosen two different senior citizens’ activity clubs and requiring several sessions for data collection due to the multi-method approach meant that participation was very unequal. This is important when planning other studies with objectives similar to this one. The reduced sample size also explains the fact that the regression model better predicted the fact of not being a volunteer than being one. Although other studies have shown that variables such as economic level, health status, and level of education favor volunteering, this information was not available and therefore could not be verified in our study.

## 10. Future Lines of Research

The quantitative data obtained displayed significant results for the subjective well-being indicators used. However, the participants in the discussion groups reported that, besides being an important source of personal satisfaction, volunteering allows them to experience productive aging, help others (by helping themselves), feel useful, and increase their self-esteem. These latter aspects would be framed within the psychological well-being tradition, according to which well-being consists of achieving or realizing individual internal motivations [48], which leads us to suggest the inclusion of a psychological well-being scale in future studies.

It would also be necessary to analyze the reasons why satisfaction with interpersonal relationships and the groups one belongs to is lower among volunteers, as it was an unexpected finding in this study.

Finally, the fact that volunteers and non-volunteers did not differ in the areas of satisfaction studied and that the means were similar in some areas suggests that further studies are needed to verify whether the mediation of third variables explains these results.

## Figures and Tables

**Table 1:** Questions raised in the questionnaire and in discussion groups.

Questions of the Questionaire		
General Question	Response Options	Specific Question/Item
Do you know any organization related to volunteering?	Yes	If so, could you tell us which one or which ones?
	No	
Do you know anyone of your age who volunteers?	Yes	If yes: Please state whether it is: □ A relative □ A neighbor □ A friend □ An acquaintance from the senior citizens’ activity club □ An acquaintance from outside the senior citizens’ activity club
	No	
Do you know what kind of work is done by older adults volunteers in the field of health?	Yes	
	No	
Have you ever volunteered?	Yes	- If yes: - Are you volunteering at the moment? - Was the volunteer work you did social, cultural, or other? - To what degree do you think that the local authorities encourage volunteering among the older adults? (1 = not at all; 5 = a lot). - What support or help for volunteering do you receive from family members/relatives of people who receive help through volunteering/friends/neighbors/other volunteers/acquaintances from the senior citizens’ activity clubs/health personnel/volunteer organizations? (1 = none; 5 = a lot). - Are you satisfied with the support received? (0 = totally dissatisfied; 10 = totally satisfied). - How satisfied are you with doing or having done volunteering? (0 = totally dissatisfied; 10 = totally satisfied). - State 2 positive and 2 negative consequences of volunteering. - To what extent do you think a volunteer should have the following characteristics? - Response scale from 0 (in total disagreement) to 5 (totally agree): being patient, being able to connect with the emotions of others, being optimistic, being able to to listen, being polite/happy/generous/a believer/supportive, having communication and conversation skills. (The items included in this question were inspired in the AEP list. The full list was not used because it was too long for the purposes of our study and sample characteristics.)
	No	
Do you think that older adults can help with health issues?	Yes	If the answer was “Yes”, they were asked in which ways they could help.
	No	
To what extent do you think that these factors can encourage older people to volunteer with other seniors in the field of health?	Scale from 0 (not at all) to 5 (a lot)	Being healthy/having medical knowledge/not having far to travel/having own car/having the support of family members/meeting older adults volunteers/having training/having time/that volunteering does not involve expenses/belonging to an older adults organization/being appreciated.
Currently, how satisfied are you with each of the following things in your life?	Personal Well-Being Index (PWI) [35,36]	This instrument includes 7 items of satisfaction with different life domains plus satisfaction with religion or spirituality, which the authors consider to be optional. In our study, we included the following 8 domains: satisfaction with your health, with your standard of living, with the things you have achieved, with how safe you feel, with the feeling of belonging to the community, with security for your future, with your relationships with other people, and with spirituality and/or religion. The reason for including this last domain has to do with the fact that the World Health Organization [37] and recent research [38,39] recognize the importance of spirituality for quality of life. The Cronbach’s alpha for the original scale lies between 0.70 and 0.85 in Australia and overseas and displays a correlation of 0.78 with the satisfaction with life scale [35]. In a study conducted with community-dwelling older adults, the PWI obtained a Cronbach’s α of 0.88 and a correlation of 0.50 with the item satisfaction with life as a whole [40]. In a more recent study with older Chilean people, the Cronbach’s α was 0.92, and the corrleation with satisfaction with life in general provided a correlation with a value of 0.766 [41]. Our sample displayed a Cronbach’s α of 0.88 and a correlation of 0.79 with satisfaction with life as a whole. The scale ranges from 0 (totally dissatisfied) to 10 (totally satisfied). The item “satisfied with feeling part of the community” was replaced with “satisfaction with the groups of people you belong to”, given that the former item has not been observed to function well in previous studies in the Spanish context [42]. The instrument was administered using the Spanish or Catalan version validated by Casas et al. [43], depending on the preference of the person being interviewed.
An item on overall life satisfaction was added, following the recommendations made by Campbell et al. [44] and the scientific consensus from that time on, according to which the exploration of satisfaction with different life domains should be completed with this item.		
Questions raised in the discussion groups:		
What do you think about volunteering in the health field? Have you ever considered volunteering in the health field? Under what conditions would you be willing to volunteer in the health field? Do you think that older people are predisposed to get involved in volunteering in the health field? Under what conditions do you think older adults would be willing to do this? Do you think that participating in volunteer work improves the well-being and health of the people who do it? What should be done to encourage many much older people to volunteer in the health field?		

**Table 2:** Personal characteristics that a volunteer must have; % of favorable responses (agree to some extent or totally agree).

Personal Characteristics	% Responses Agree to Some Extent or Totally Agree
Being polite	89
Being optimistic	85.9
Knowing how to listen	85.7
Being supportive	85.3
Knowing how to connect with the emotions of others	80.9
Being patient	79.2
Having communication and conversation skills	76.8
Being a good person	76.8
Being generous	76
Being calm	71.8
Being happy	65.2
Having a faith	27.3

**Table 3:** Factors that facilitate volunteering. Scale from 0 to 5 (0 = not at all and 5 = a lot).

	Mean	SD
Being in good health	4.29	1.013
Having time	4.25	1.005
Knowing older adults volunteers	3.78	1.256
Having the support of family members	3.77	1.377
Not having to travel far	3.68	1.277
Having the necessary training	3.53	1.197
Volunteering not involving expenses	3.52	1.533
Being valued for the work you do	3.03	1.507
Belonging to an older adults organization	3.03	1.449
Having own car	2.87	1.485
Having medical knowledge	2.87	1.338

**Table 4:** Satisfaction with the different satisfaction domains included in the PWI. Scale from 0 to 10 (0 = totally dissatisfied and 10 = totally satisfied).

Well-Being Domains	General Sample	Non-Volunteers	Volunteers	F	*p*	η_p_^2^
Overall life satisfaction	7.39	6.76	8.12	0.752	0.010	0.110
Interpersonal relationships	7.39	7.42	7.20	0.123	0.727	0.002
Achievements	7.17	6.85	7.38	0.774	0.382	0.013
Groups you belong to	7.10	7.35	6.81	0.950	0.334	0.017
Perceived present safety	6.92	6.72	6.86	0.057	0.812	0.001
Standard of living	6.72	6.47	6.83	0.366	0.548	0.006
Health	6.47	6.22	6.77	0.761	0.386	0.012
Future security	6.41	6.26	6.58	0.234	0.630	0.004

**Table 5:** Binary logistic regression model fit.

–2 LL	χ^2^	df	P	R^2^ Cox y Snell	R^2^ Nagelkerke
52.271	19.667	8	0.012	0.310	0.417

**Table 6:** Classification of sub-groups included in the model.

% Sample Included	% General Prediction	% Non-Voluntary Prediction	% Voluntary Prediction
62.4	71.7	83.9	54.5

**Table 7:** Correlation of variables included in the binary logistic regression model with the variable having done or doing volunteer work as a dependent variable.

Variables Included in the Regression	Β	t	Odds Ratio	*p*-Value
Overall life satisfaction	1.116	6.403	3.052	0.011
Satisfaction with groups you belong to	−0.970	5.949	0.379	0.015
Satisfaction with perceived present safety	−0.530	2.520	0.589	0.112
Satisfaction with achievements in life	0.553	2.445	1.739	0.118
Satisfaction with health	0.303	1.014	1.354	0.314
Satisfaction with standard of living	−0.351	0.985	0.704	0.321
Satisfaction with interpersonal relationships	−0.225	0.496	0.799	0.481
Satisfaction with future security	0.124	0.178	1.132	0.673

**Table 8:** Coincidences and discrepancies in the opinions between the participants from the two senior citizens’ activity clubs.

Coincidences	Discrepancies
- Important characteristics that volunteers should have relate to characteristics of “extraversion and agreeableness”. - Volunteering requires training and vocation. Caring for a family member at home is obligatory and involves greater sacrifice. - Methods need to be found to promote and reward volunteering behaviors. - Volunteering brings benefits in the form of self-esteem and well-being.	- On information and resources regarding volunteering. In Horta, it was considered that there are many volunteer associations, and that those who are interested in this type of activity know about it. In Besalú, it was considered that there is not enough information about volunteering and the tasks that can be carried out, and that there are not enough resources.

## Data Availability

Not applicable.
